# Identification of novel *PKD1* and *PKD2* mutations in a Chinese population with autosomal dominant polycystic kidney disease

**DOI:** 10.1038/srep17468

**Published:** 2015-12-03

**Authors:** Bei Liu, Song-Chang Chen, Yan-Mei Yang, Kai Yan, Ye-Qing Qian, Jun-Yu Zhang, Yu-Ting Hu, Min-Yue Dong, Fan Jin, He-Feng Huang, Chen-Ming Xu

**Affiliations:** 1 Women’s Hospital School of Medicine Zhejiang University, Hangzhou 310006, P. R. China; 2 Key Laboratory of Reproductive Genetics (Zhejiang University), Ministry of Education, Hangzhou 310006, P. R. China; 3 Institute of Embryo-Fetal Original Adult Disease Affiliated to Shanghai Jiao Tong University School of Medicine, Shanghai 200030, P.R. China; 4 The International Peace Maternity & Child Health Hospital Affiliated to Shanghai Jiao Tong University School of Medicine, Shanghai 200030, P. R. China

## Abstract

Autosomal dominant polycystic kidney disease (ADPKD) is one of the most frequently inherited renal diseases caused by mutations in *PKD1* and *PKD2*. We performed mutational analyses of *PKD* genes in 49 unrelated patients using direct PCR-sequencing and multiplex ligation-dependent probe amplification (MLPA) for *PKD1* and *PKD2*. RT-PCR analysis was also performed in a family with a novel *PKD2* splicing mutation. Disease-causing mutations were identified in 44 (89.8%) of the patients: 42 (95.5%) of the patients showed mutations in *PKD1*, and 2 (4.5%) showed mutations in *PKD2*. Ten nonsense, 17 frameshift, 4 splicing and one in-frame mutation were found in 32 of the patients. Large rearrangements were found in 3 patients, and missense mutations were found in 9 patients. Approximately 61.4% (27/44) of the mutations are first reported with a known mutation rate of 38.6%. RNA analysis of a novel *PKD2* mutation (c.595_595 + 14delGGTAAGAGCGCGCGA) suggested monoallelic expression of the wild-type allele. Furthermore, patients with *PKD1*-truncating mutations reached end-stage renal disease (ESRD) earlier than patients with non-truncating mutations (47 ± 3.522 years vs. 59 ± 11.687 years, *P* = 0.016). The mutation screening of *PKD* genes in Chinese ADPKD patients will enrich our mutation database and significantly contribute to improve genetic counselling for ADPKD patients.

Autosomal dominant polycystic kidney disease (ADPKD) is one of the most frequently inherited renal diseases worldwide with an estimated incidence of 1:400 to 1:1,000 and is characterized by bi-lateral renal cysts in the liver, seminal vesicles, pancreas and arachnoid membrane, as well as extra-kidney abnormalities^1^. ADPKD is a common kidney disorder in China, affecting more than 1.3 million Chinese individuals. Approximately 10–20% of ADPKD children are hypertensive, and the majority of adults have hypertension with normal kidney function^2^. Approximately 50% of individuals with ADPKD will progress to end-stage renal disease (ESRD) by the age of 60 years, and these patients account for 5–8% of the renal replacement population^3^.

ADPKD is a heterogeneous monogenic disorder resulting from mutations in two genes: *PKD1* and *PKD2*. The *PKD1* gene (OMIM 601313), located in chromosome region 16p13.3, consists of 46 exons with an open reading frame of 12,912 bp that encodes the 4,303-amino acid peptide polycystin-1 (PC1)^4^. Exons 1–33 of *PKD1* are duplicated approximately six times at the homologous genes (HGs), which has made the genetic analysis of *PKD1* challenging^5^. *PKD2* (MIM 173910, chromosome region 4q21-22) is a single-copy gene including 15 exons with a 2,907-bp coding sequence and is predicted to encode a 968-amino acid peptide called polycystin-2 (PC2)^6^. PC1 and PC2 act as flow-dependent mechanosensors in renal primary cilium that regulate the differentiated state of tubular epithelial cells^7^. Clinical data show that mutations in *PKD1* and *PKD2* account for 85% and 15% of all cases of ADPKD, respectively^8^. Compared to *PKD2* mutations (where the average age of ESRD onset is 74 years), *PKD1* mutations (where the average age of ESRD onset is 54.3 years) are associated with the more serious form of the disease^9^.

Diagnosis of ADPKD is performed mainly by renal ultrasound, computed tomography (CT) or magnetic resonance imaging (MRI) but cannot exclude the disease in at-risk individuals until the age of 40 years, especially in families with mutations in *PKD2*^10^. Molecular diagnostics play a significant role in confirming a definite diagnosis, especially in young renal donors, patients with a negative family history, individuals with early onset ADPKD or atypical symptoms and for subjects with affected relatives^11^. There is no hotspot mutation for *PKD1* or *PKD2*, indicating that mutations are usually unique to a single family and are highly variable^12^. The structural complexity of *PKD1* and the high allelic heterogeneity of *PKD* genes make clinical molecular diagnostics difficult^13^. Although a few studies on novel mutations in the *PKD1* and *PKD2* genes in Chinese patients have been carried out with different methods, including PCR-single-strand conformation polymorphism (PCR-SSCP)^14^, denaturing high-performance liquid chromatography (DHPLC)^15^ and next-generation sequencing (NGS)^16^, direct sequencing is undoubtedly the gold standard for accurately identifying the majority of *PKD* mutations^17^. Multiplex ligation-dependent probe amplification (MLPA) was developed to detect large genomic rearrangements in *PKD* genes that cannot be detected by sequencing^18^.

Analysis of the pathogenicity of variants of an uncertain significance plays an important role in the molecular diagnosis of ADPKD because of the high level of genetic variation found in the *PKD1* gene^19^. So far, approximately 2,322 sequence variants of *PKD1* and 278 sequence variants of *PKD2* have been reported in the Autosomal Dominant Polycystic Kidney Disease Mutation Database (PKDB, http://pkdb.mayo.edu; January 2015). The Human Gene Mutation Database (HGMD) has recorded 1,177 sequence variants of *PKD1* and 211 sequence variants of the *PKD2* gene (http://www.hgmd.cf.ac.uk). However, mutation data for *PKD* genes from different populations would provide a better interpretation of genetic testing results.

In the present study, we performed long-range PCR (LR-PCR) followed by nested PCR and MLPA of *PKD1* and *PKD2* in 49 Chinese patients with a definite diagnosis of ADPKD. A group of novel mutations in *PKD1* and *PKD2* is described in this paper. All mutation data detected will contribute to better diagnostics and genetic counselling in a clinical setting.

## Results

**Figure 1:**
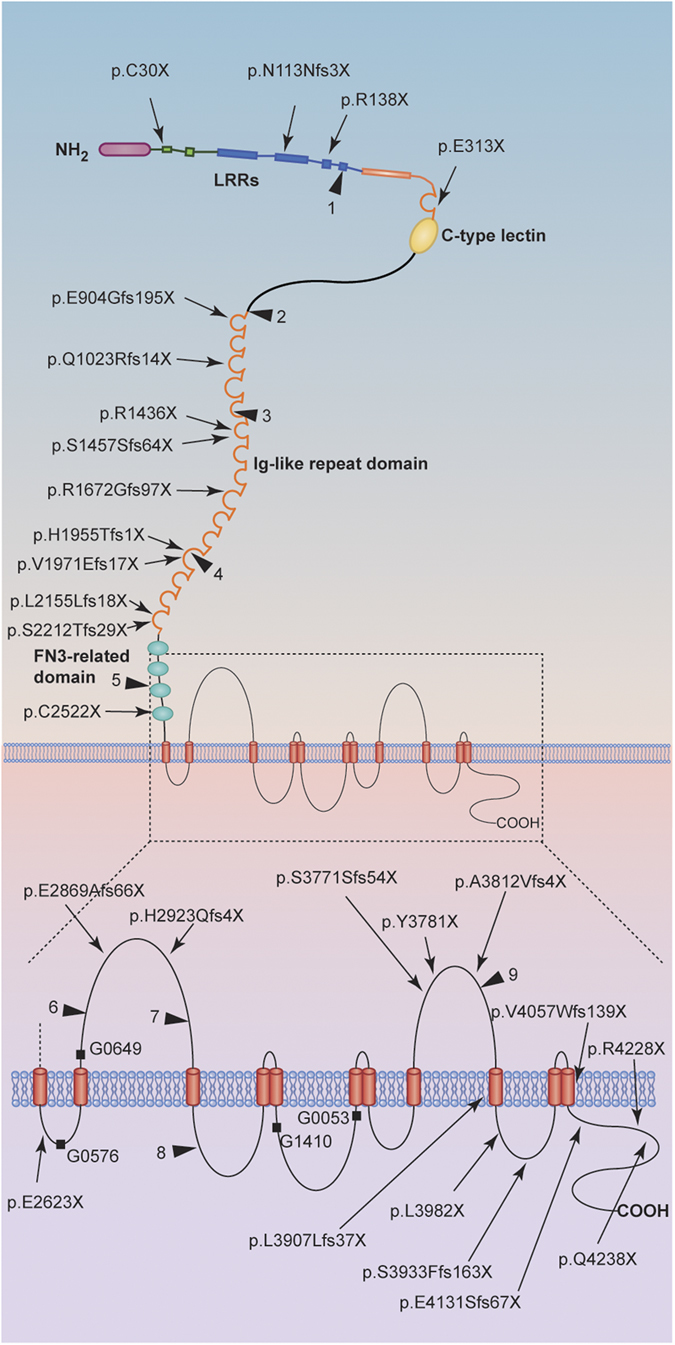
Schematic representation of polycystin-1, representing the location of mutations found in this study or previous reports (adapted by permission from Macmillan Publishers Ltd: [NATURE GENETICS], Hughes*et al.*1995^4^). Nonsense mutations and frameshift mutations are indicated with arrows. The positions of in-frame deletions and splicing changes are indicated with boxes (G0576: c.8017-2-1delAG; G0649: c.8157-8159delCAC; G1410: c.10220 + 2T > C; G0053: c.10617 + 1delG). Likely pathogenic mutations are indicated with triangles (1–9: see Table 3 for details).

We performed complete mutational analysis by direct sequencing and MLPA analysis of *PKD1* and *PKD2* in 49 unrelated patients with the diagnosis of ADPKD obtained by ultrasound. Thirty-two definitely pathogenic variants and 12 likely pathogenic variants (42 variants in *PKD1* and 2 in *PKD2*) were found in 44 patients (Table 1), resulting in a variant detection rate of 89.8% (44/49). The variants we found represent 61.4% (27/44) novel variants and 38.6% (17/44) known variants. The remaining 5 patients (10.2%) were classified with an undetermined genotype either because no variant was detected or the identified variant was considered to be nonpathogenic. Figure 1 shows the distribution of definitely pathogenic and likely pathogenic variants found in the *PKD1* gene. We further confirmed that there are no variant hotspots in *PKD1* gene.

### Definite pathogenic mutations

Definite pathogenic mutations were found in 32 of the families and included 10 nonsense mutations, 17 frameshift mutations, 2 splicing mutations, and 3 large rearrangements. These disease-causing mutations are shown in Table 2. The percentage of our ADPKD patients without a family history was 12.2% (6/49). We analysed *PKD1* and *PKD2* in the parents of six probands with no family history of ADPKD and found that in five instances, the pathogenic mutations occurred *de novo* in the probands. No pathogenic mutation of *PKD1* or *PKD2* was found in the other patient without a family history ADPKD.

### RT-PCR analysis of *PKD2* mRNA in patient G0904

A novel splicing mutation in *PKD2* (c.595_595 + 14delGGTAAGAGCGCGCGA) was found in patient G0904 and was predicted to affect the splice site of the gene. The predicted absence of *PKD2* exon 2 would produce a premature termination codon (PTC) downstream in exon 3 and therefore could not escape nonsense-mediated mRNA decay (NMD)^20^,^21^. To evaluate the influence of the c.595_595 + 14del, RT-PCR analysis of total RNA extracted from peripheral blood mononuclear cells (PBMCs) from patient G0904 and a healthy control was performed using the primers *PKD2*-E1F/*PKD2*-E2R and *PKD2*-E1F/*PKD2*-E4R. It is worth noting that we could not detect aberrant *PKD2* transcripts in patient G0904 (Fig. S1A). However, heterozygosity of rs2728118 (c.420G > A) in exon 1 of *PKD2* was only found in the genomic DNA (GA) and not in the cDNA of the patient (AA) (Fig. S1C). rs2728118 was inherited from the patient’s father, and the c.595_595 + 14del mutation was inherited from the patient’s mother (Fig. S1B). rs2728118 is located upstream of the c.595_595 + 14del mutation and is distributed in *trans* on the chromosome. Further quantitative PCR (qPCR) analysis indicated that the patient *PKD2* mRNA level was reduced to 55.97 ± 1.78% (*P* = 5.44 × 10^−5^) compared to the normalized control mRNA level (Fig. S1D). These results suggested monoallelic expression of the wild-type allele, inheritance form the patient’s father and nonsense-mediated mRNA decay (NMD) of the aberrantly spliced *PKD2* transcripts.

### Likely pathogenic mutations

**Figure 2:**
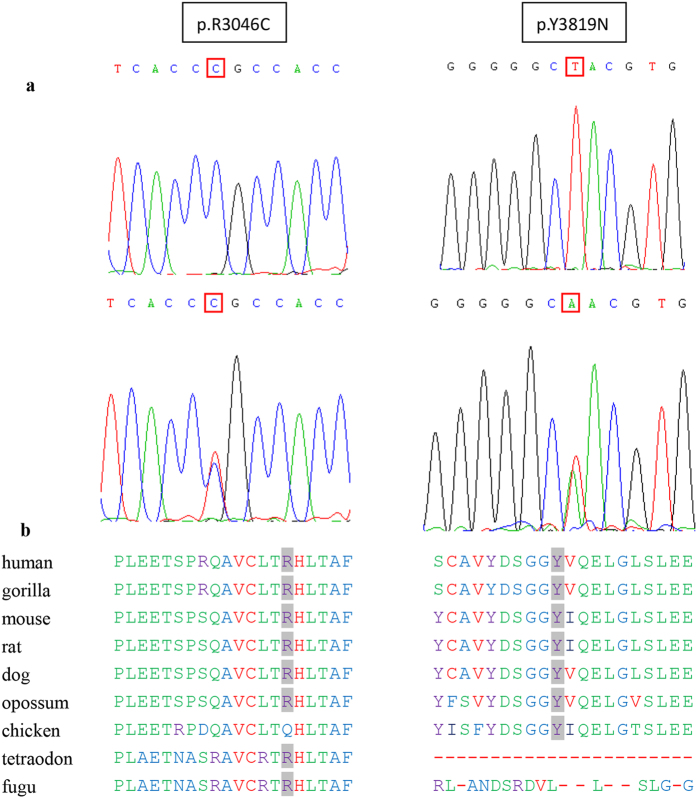
Two *PKD1* missense mutations (p.R3046C and p.Y3819N) coexisted in a Chinese patient with ADPKD. (**a**) The sequencing pattern of p.R3046C and p.Y3819N in patient G0241. Both sequences are in the forward direction. The bottom line corresponds to the mutated sequence; the upper line represents the wild-type sequence. (**b**) Multiple sequence alignments for the *PKD1* variant changes p.R3046C and p.Y3819N.

A total of 22 unclassified variants and 12 previously reported polymorphisms (Table S1) were detected in our patients. We evaluated the pathogenic potential of the unclassified variants using web-based prediction programs. The results of the evaluation are presented in Table 3. Twelve likely pathogenic mutations (11 *PKD1* mutations and 1 *PKD2* missense mutation) were identified in 12 patients. Among these mutations, one small novel in-frame mutation in *PKD1* (c.8157-8159delCAC) showed high evolutionary conservation, and two novel splicing variants in *PKD1* (c.10220 + 2T > C and c.10617-1delG) were predicted to affect the splice site and were found to segregate with the disease in affected families. Therefore, these three mutations in *PKD1* are highly likely to be pathogenic mutations. Two novel substitutions *PKD1* (p.R3046C and p.Y3819N) coexisted in patient G0241 and were predicted *in silico* to be likely pathogenic and likely polymorphic, respectively. Amino acid multi-alignments demonstrated that the positions of the mutations are highly conserved across species for both p.R3046C and p.Y3819N (Fig. 2). Therefore, we speculate that p.R3046C and p.Y3819N are hypomorphic alleles. Segregation of the two variants with the disease was not validated due to the lack of blood samples from the families.

### Large deletion mutation

To identify large deletions or duplication mutations that cannot be detected by Sanger sequencing, we performed a copy number analysis of the probands without pathogenic mutations in the *PKD* genes (Fig. S2). Using MLPA, three large deletions of the *PKD1* gene were found in patients G0677, G0018 and G1800. Deletion of *PKD1* exon 1 (relative peak ratio 0.52) was found in patient G0677, a 45-year-old man, and a similar result was found in his affected family members. Deletion of *PKD1* exon 21 (relative peak ratio 0.51) was identified in patient G1800 and his affected mother. Patient G1800 is 26 years old and was found to have bilateral renal cysts at approximately 18 years of age. The renal function of patient G1800 is well controlled except for slight hypertension. DNA sequence analysis of exons 1 and 21 demonstrated the absence of single-base mutations under the oligonucleotide probe. Patient G0018, who is 20 years old and without a family history of ADPKD, showed a deletion for probes 1 to 30. Large deletion mutations segregated with the disease in all of the affected family members tested, although q-PCR confirmation was not performed at the nucleotide level due to the sequence complexity of the *PKD1* locus.

### Genotype/phenotype correlation

**Figure 3:**
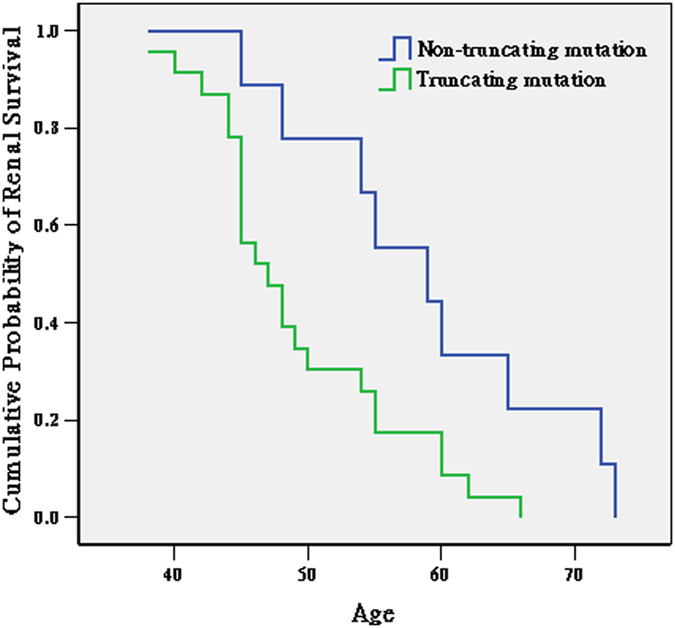
Kaplan-Meier survival curves showing the age of ESRD onset for patients with truncating mutations (n = 23) and patients with non-truncating mutations (n = 9) in *PKD1* in our study cohort (*P* = 0.016, log-rank test).

Of the 49 probands, 32 had already reached ESRD (Table 4). We therefore performed a Kaplan-Meier survival curve analysis to investigate whether the type of *PKD1* mutation (non-truncating mutations including missense and small in-frame mutations vs. truncating mutations) influenced the age of ESRD onset. As shown in Fig. 3, the age of ESRD onset in patients with *PKD1*-truncating mutations (n = 23) was earlier than that of the patients with non-truncating *PKD1* mutations (n = 9) (log-rank test, *P* = 0.016). The median age of ESRD onset in the patients with *PKD1*-truncating mutations and patients with non-truncating mutations was 47 years (95% CI, 47 ± 3.522 years) and 59 years (95% CI, 59 ± 11.687 years), respectively.

## Discussion

To date, 2,322 pathogenic mutations for *PKD1* and 278 for *PKD2* have been reported in the PKDB. In our study, we analysed 49 ADPKD families using DNA sequencing and MLPA with a mutation detection rate of 89.8%. Among the 44 mutations we found, 61.4% (27/44) are novel mutations and 38.6% (17/44) are known mutations. The 44 pathogenic mutations we identified occurred in unrelated patients, suggesting that each patient had a unique pathogenic mutation. Our findings are consistent with previous studies showing that less than 2% of unrelated ADPKD-affected families carry the same mutation^22^. Our results indicate that no mutation hotspot exists in either *PKD1* or *PKD2*. Therefore, a complete mutation analysis of *PKD1* and *PKD2* is needed for Chinese patients with ADPKD who require a genetic diagnosis. The 27 novel mutations we found in the Chinese population will enrich the *PKD* mutation database and significantly contribute to the genetic counselling of ADPKD patients.

Patients with *PKD2* mutations typically develop ESRD two decades later than those with mutations in *PKD1*^9^. Therefore, it is of prognostic value to determine the location of the mutation in an affected family (*PKD1* or *PKD2*). The mutational distribution in the *PKD2* gene could not be determined because only two exonic mutations were found. The mutation detection rate of *PKD2* (4.1%) in our study was much lower than the average percentage (15%)^8^. This difference may be due to the low number of patients analysed in our study compared to previous studies by Rossetti *et al.*

Previous studies have shown that the mutation type and location in *PKD1* may influence renal survival^23^. We detected a total of 44 pathogenic variations in the *PKD1* gene; of these, 9 (20.9%) are located in exon 15, which is consistent with the findings recorded in the PKDB (277/1,272). This region corresponds to the junction of the *PKD* repeats and the REJ domain of the resultant protein. In our study, 78.6% of the mutations in *PKD1* were predicted to truncate the protein, including frameshift mutations, nonsense mutations, splicing mutations, and large deletions. The high frequency of these mutations is in concordance with recent results from Cornec-Le Gall *et al.*, who showed that approximately two thirds of *PKD1* mutation-positive pedigrees carry truncating mutations^24^. Cornec-Le Gall *et al.* reported that carriers of a *PKD1*-truncating mutation have a significantly earlier age of ESRD onset than patients with a non-truncating mutation (55 years vs. 67 years). Our data support the view that a more severe phenotype can be expected in patients with a *PKD1*-truncating mutation. The exception was patient G0599, who carried the known missense mutation p.C155Y but also had received a right renal transplant at 26 years old.

Two novel *PKD1* mutations (p.R3046C and p.Y3819N) were found in a female patient with ADPKD, and it is probable that both mutations are hypomorphic alleles. However, DNA samples from the parents of this patient were not available. Moreover, the likely pathogenic mutations identified in our study should be confirmed in future studies including additional ADPKD families.

Large genomic rearrangements account for approximately 6.8% of the pathogenic mutations in the *PKD1* gene and an even smaller percentage of *PKD2* gene mutations^18^. In our study, we identified three large deletions: one involving deletion of exon 21, one involving deletion of exons 1 to 30, and a large deletion of exon 1^25^. Our data are consistent with previously reported data.

The high level of allelic heterogeneity in both the *PKD1* and *PKD2* genes and the prevalence of private mutations in ADPKD patients imply that there is a high frequency of *de novo* mutations in this disease. Indeed, approximately 10% of adult ADPKD patients do not have a family history of the disease^26^. The percentage of our ADPKD patients without a family history of disease was 12.2%, which is consistent with previously reported results. Five different pathogenic mutations occurred *de novo* in probands without a family history of ADPKD.

Pathogenic mutations were not found in 10.2% of our 49 unrelated patients, a result in accordance with those reported in a previous CRISP study (10.9%)^8^. This finding may be because mutations occur within deep intronic regions, as well as in promoters and other distantly located regulatory regions not covered by the current exon-based sequencing method. Alternatively, some of the missense mutations that are classified as nonpathogenic mutations by the prediction system may represent hypomorphic alleles. Such variants alone may result in only mild cystic disease, but two such variants in trans may cause disease^27^,^28^. Additionally, we only screened *PKD1* and *PKD2* mutations and therefore cannot exclude the existence of other mutations that might contribute to the cystic phenotype, such as those in *HNF1b*, *PRKCSH*, *SEC63* or *PKHD1*^29^. Furthermore, mosaicism may influence the genotype and phenotype of ADPKD; however, this condition is usually not detected in screenings, and a significant proportion of *de novo* mosaic mutations may be missed^30^,^31^. The likely pathogenic mutations identified in our study need to be confirmed in future studies with additional ADPKD families.

## Conclusions

In our study, 27 novel pathogenic mutations in the *PKD* genes were detected in 49 Chinese individuals. A novel splicing mutation in *PKD2* (c.595_595 + 14delGGTAAGAGCGCGCGA) was confirmed to be definitely pathogenic. Patients carrying *PKD1* mutations, especially those with truncating mutations, could have a more rapidly progressive disease than those with non-truncating mutations. Our study will enrich the mutation database of *PKD* and significantly contribute to the genetic counselling of ADPKD patients.

## Methods

### Patients

A total of 49 unrelated patients were enrolled from the Women’s Hospital School of Medicine Zhejiang University from October 2010 to December 2013. Patients were definitively diagnosed with ADPKD based on the criteria recommended by Ravine D *et al.*^32^. All patients provided written informed consent, and their family and medical histories were recorded. The general clinical data of the probands are summarized in Table 5. Peripheral blood samples were collected from all probands and their family members when possible. The study was performed with the approval of the Ethics Committee of the Women’s Hospital School of Medicine Zhejiang University. The study was conducted in adherence to the Declaration of Helsinki.

### Mutation analysis of *PKD1* and *PKD2*

Genomic DNA was extracted from peripheral blood samples using the QIAGEN spin columns on a QIACube (QIAGEN GmbH) according to the manufacturer’s instructions. The mutational screening of *PKD1* and *PKD2* via Sanger sequencing was carried out in the 49 probands. LR-PCR followed by nested PCR was adopted for the mutational analysis of the *PKD1* gene^5^. The duplicated region of *PKD1* was amplified in eight specific long fragments by LR-PCR (exon 1, 2–7, 8–12, 13–15, 15–21, 22, 23–28 and 29–34)^33^ using TaKaRa LA Taq TM (TaKaRa Bio Inc.). Exons 1–34 of *PKD1* were then amplified by nested PCR with these LR templates, and exons 35–46 were directly PCR amplified and sequenced in both directions. Exons 1–15 of *PKD2* including the adjacent 30–50 bp intronic sequence was amplified from genomic DNA according to a previous report with little modification^34^. PCR amplification primers for the various LR-PCR fragments are provided in Supplemental Table S1.

### MLPA

MLPA analysis was performed with a SALSA MLPA KIT P351-B1/P352-B1 *PKD1-PKD2* kit (MRC-Holland, Amsterdam, the Netherlands) according to the manufacturer’s instructions. The kit covers *PKD1* probes for exons 37 to 46, its upstream regulatory sequences, all *PKD2* exons and three exons of the *TSC2* gene adjacent to the *PKD1* gene^35^. The result of our MLPA analysis was scanned using the ABI PRISM® 3100 Genetic Analyzer (Applied Biosystems). The data were analysed using the Coffalyser MLPA analysis tool (MRC-Holland, Amsterdam, the Netherlands).

### RT-PCR analysis of *PKD2* mRNA in PBMCs

Total RNA from PBMCs was extracted and reverse transcribed from patient G0904 and a healthy control. RT-PCR was performed with primers PKD2-E1F/PKD2-E2R and PKD2-E1F/PKD2-E4R. qPCR was performed with primers PKD2-E13F/PKD2-E14R, and the results were normalized to glyceraldehyde-3-phosphate dehydrogenase (*GAPDH*), according to the method described previously^36^. RT-PCR and qPCR primers are listed in Table S3.

### Data analysis and sequence variation classification

Mutations of *PKD* genes were analysed using Mutation Surveyor® software. Nucleotide changes were nominated according to the NCBI reference sequences of *PKD1* (NM_001009944.2) and *PKD2* (NM_000297.3). The HGMD (http://www.hgmd.cf.ac.uk), the Exome Sequencing Project (http://evs.gs.washington.edu/EVS) and PKDB (http://pkdb.mayo.edu) were checked for previously reported sequence changes. Novel mutations in this study were assessed for their pathogenic potential. Nonsense or frameshift variants resulting in a PTC were identified to be definitely pathogenic. The pathogenicity of missense variants was computationally evaluated with the SIFT, PolyPhen2 and AlignGVGD prediction programs by analysing interspecies sequence variations^37^,^38^,^39^. The NetGene2 (http://www.cbs.dtu.dk/services/NetGene2/)^40^ and Human Splicing Finder (HSF) (http://www.umd.be/HSF/)^41^ software were used to evaluate the splice site mutations. All variations analysed by these web-based software programs were finally sorted into four categories: likely pathogenic, indeterminate, likely polymorphic and polymorphic. Only gene variations that were predicted to be damaging by SIFT, PolyPhen-2 and AlignGVGD were considered to be “likely pathogenic”, as long as no other definite mutation was found in the same patient. If a definite mutation coexisted with a damaging missense mutation in the same patient, the missense mutation was considered to be “indeterminate”. Similarly, only the variations that were scored as benign by all the software programs were considered to be “polymorphic”. Otherwise, the mutations were classified as “likely polymorphic”. Furthermore, pedigree co-segregation analysis of the potential pathogenic mutations in *PKD* genes was examined in all available members of the probands’ families (including healthy individuals). Likely pathogenic missense or splice site variants would segregate within the affected family.

### Statistical analysis

Cumulative renal survival curves were generated using the Kaplan–Meier method and compared using the log-rank test. *P* < 0.05 was considered to be statistically significant. All analyses were performed using SPSS 17.0.

## Additional Information

**How to cite this article**: Liu, B. *et al.* Identification of novel *PKD1* and *PKD2* mutations in Chinese population with autosomal dominant polycystic kidney disease. *Sci. Rep.* **5**, 17468; doi: 10.1038/srep17468 (2015).

## Supplementary Material

Supplementary Information

## Figures and Tables

**Table 1:** Characteristics of the detected mutations.

Description	*PKD1*	*PKD2*	Total
Pathogenic	31	1	32
Likely pathogenic	11	1	12
Frameshift	17	0	17
Nonsense	10	0	10
Splicing	3	1	4
Large deletion	3	0	3
In-frame deletion	1	0	1
Missense	8	1	9
Known mutations	17	0	17 (38.6%)
Novel mutations	25	2	27 (61.4%)
Total mutations detected	42 (95.5%)	2 (4.5%)	44

**Table 2:** Definite pathogenic mutations in *PKD1* and *PKD2* found in this study.

Family No.	Exon/intron	cDNA change	Protein change	Mutation Type	Proband No.	Family history	Previous description
***PKD1***							
28	1	c.90C > A	p.Cys30Ter	Nonsense	G0586	Yes	Novel
17	3	c.338-341delATTT	p.Asn113AsnfsX3	Frameshift	G0211	Yes	Novel
13	4	c.412C > T	p.Arg138Ter	Nonsense	G1739	Yes	PKDB
20	5	c.937G > T	p.Glu313Ter	Nonsense	G0214	Yes	PKDB
51	11	c.2711-2712delAG	p.Glu904GlyfsX195	Frameshift	G1604	Yes	Novel
42	13	c.3068delA	p.Gln1023ArgfsX14	Frameshift	G0463	Yes	Novel
1	15	c.4306C > T	p.Arg1436Ter	Nonsense	G0256	Yes	PKDB
9	15	c.4369-4370delTC	p.Ser1457SerfsX64	Frameshift	G0315	Yes	PKDB
41	15	c.5014-5015delAG	p.Arg1672GlyfsX97	Frameshift	G0262	Yes	PKDB
32	15	c.5863delC	p.His1955ThrfsX1	Frameshift	G0679	Yes	Novel
48	15	c.5912-5913delTG	p.Val1971GlufsX18	Frameshift	G1052	Yes	PKDB
37	15	c.6465-6466delGC	p.Leu2155LeufsX18	Frameshift	G1103	Yes	Novel
33	15	c.6635delG	p.Ser2212ThrfsX29	Frameshift	G0680	Yes	Novel
5	19	c.7566C > A	p.Cys2522Ter	Nonsense	G0246	No^a^	Novel
49	21	c.7867G > T	p.Glu2623Ter	Nonsense	G1436	No^a^	Novel
27	IVS21	c.8017-2-1delAG	p.Gly2673fs	Splice	G0576	Yes	PKDB
11	23	c.8606-8607delAG	p.Gln2869AlafsX66	Frameshift	G0111	Yes	PKDB
19	24	c.8495-8496insA	p.His2932GlnfsX4	Frameshift	G0213	Yes	Novel
39	40	c.11313delG	p.Ser3771SerfsX54	Frameshift	G0017	No^a^	Novel
14	40	c.11343C > A	p.Tyr3781Ter	Nonsense	G0054	Yes	16
21	41	c.11433-11439dupCGTGTAT	p.Ala3812ValfsX4	Frameshift	G0224	No^a^	Novel
15	43	c.11719-11720dupCT	p.Leu3907LeufsX37	Frameshift	G0092	Yes	Novel
46	43	c.11944C > T	p.Gln3982Ter	Nonsense	G0863	Yes	PKDB
45	43	c.11977-11978insT	p.Ser3993PhefsX163	Frameshift	G0858	Yes	Novel
34	45	c.12169delG	p.Val4057TrpfsX139	Frameshift	G0865	Yes	Novel
3	45	c.12391delG	p.Glu4131SerfsX67	Frameshift	G0251	Yes	Novel
50	46	c.12682C > T	p.Arg4228Ter	Nonsense	G1543	Yes	PKDB
36	46	c.12712C > T	p.Gln4238Ter	Nonsense	G1021	Yes	PKDB
30	1	EX1del		Large deletion	G0677	Yes	25
52	21	EX21del		Large deletion	G0121	Yes	Novel
40	1–30	EX1-30del		Large deletion	G0018	No^a^	Novel
***PKD2***							
44	IVS1	c.595_595 + 14delGGTAAGAGCGCGCGA		Splice	G0904	Yes	Novel

PKDB, Polycystic Kidney Mutation Database.

^a^de novo mutation.

**Table 3:** Evaluation of the pathogenic potential of variants.

Exon/Intron	cDNA change	Protein change	Known/Novel	Co-occurrence	SIFT MG	Polyphen-2 MG	AlignGVGD MG^c^	Proband ID	Family No.	Segregation ( + /−)	Final Prediction
*PKD1*											
4	c.464G > A	p.Cys155Tyr	PKDB^a^		B	N	N	G0599	43	+	B
8	c.1673C > T	p.Thr558Met	Novel	p.Ser3771SerfsX54	N	N	N	G0017	39	NA	E
11	c.2534T > C	p.Leu845Ser	PKDB^a^	p.Val951Ala	B	B	B	G0247	4	+	B
11	c.2852T > C	p.Val951Ala	Novel	p.Leu845Ser	N	B	B	G0247	4	—	D
14	c.3193C > A	p.His1065Pro	Novel	p.Asn3188Lys	N	C	N	G0122	8	—	D
15	c.3548C > G	p.Ser1183Trp	Novel	p.Ser1457SerfsX64	N	B	N	G0315	9	NA	E
15	c.3955G > A	p.Gly1319Arg	16 a		B	B	B	G0210	16	+	B
15	c.5824C > T	p.Arg1942Cys	Novel		C	C	C	G0240	7	+	C
15	c.6496C > T	p.Arg2166Cys	PKDB^b^	p.Thr2720del	C	B	I	G0001, G0649	12,44	NA —	I
17	c.7132G > A	p.Val2378Met	Novel	p.Val1971GlufsX17	N	B	N	G1052	48	—	E
18	c.7214G > T	p.Trp2405Leu	Novel		B	C	C	G0255	2	+	C
22	c.8157—8159delCAC	p.Thr2720del	Novel	p.Arg2166Cys				G0649	44	+	B
23	c.8294G > A	p.Arg2765His	Novel		N	N	N	G0676	56	NA	E
23	c.8311G > A	p.Glu2771Lys	PKDB^a^		C	B	C	G0212	18	+	B
25	c.9136C > T	p.Arg3046Cys	Novel	p.Tyr3819Asn	B	C	C	G0241	6	NA	C
27	c.9564C > G	p.Asn3188Lys	Novel	p.His1065Pro	B	B	B	G0122	8	+	C
IVS32	c.10220+2T > C		Novel	p.Thr4018Ile	Possibly affecting the splice site	G1410	38	+	B		
IVS35	c.10618+1delG		Novel		Possibly affecting the splice site	G0053	10	+	B		
41	c.11455T > A	p.Tyr3819Asn	Novel	p.Arg3046Cys	B	C	N	G0241	6	NA	D
44	c.12053C > T	p.Thr4018Ile	Novel	c.10220 + 2T > C	C	B	I	G1410	38	—	I
*PKD2*											
1	c.17G > A	p.Arg6His	Novel	Exon1del	B	N	N	G0677	30	—	E
8	c.1796G > A	p.Gly599Asp	Novel		B	C	B	G0976	47	+	C

Abbreviations: ID, identification; NA, not analysed; MG, mutation group; PKDB, PKD mutation database; B: highly likely pathogenic; C: likely pathogenic; I: indeterminate; N: neutral; D: likely polymorphic; E: polymorphic.

^a^The mutation has been previously classified to be “highly likely pathogenic”.

^b^The mutation has been previously classified to be “indeterminate”.

^c^Polycystin orthologue alignment (human, mouse, rat, chicken, *X. tropicalis*, fugu, dog, opossum and tetraodon).

**Table 4:** Age of ESRD onset in families with *PKD1* gene mutations.

Family No.	cDNA change	Protein change	Age of ESRD in the family (M-male, F-female, years)
30	EXIdel		M45, F55
17	c.338-341delATTT	p.Asn113AsnfsX3	M45
20	c.937G > T	p.Glu313Ter	F44, F47
51	c.2711-2712delAG	p.Glu904GlyfsX195	M45, F50
32	c.5863delC	p.His1955ThrfsX1	F48
48	c.5912-5913delTG	p.Val1971GlufsX17	F40, F44
33	c.6635delG	p.Ser2212ThrfsX29	F38, F42
44	c.8157-8159delCAC	p.Thr2720del	F54
11	c.8606-8607delAG	p.Gln2869AlafsX66	F49, F45
10	c.10618 + 1delG	Probable splice defect	F45
14	c.11343C > A	p.Tyr3781Ter	F62
46	c.11944C > T	p.Gln3982Ter	F60
45	c.11977-11978insT	p.Ser3993PhefsX163	M55
3	c.12391delG	p.Glu4131SerfsX67	F46
50	c.12682C > T	p.Arg4228Ter	M60, F66
36	c.12712C > T	p.Gln4238Ter	M55
43	c.464G > A	p.Cys155Tyr	M26
4	c.2534T > C	p.Leu845Ser	M59
16	c.3955G > A	p.Gly1319Arg	M65
7	c.5824C > T	p.Arg1942Cys	F55
2	c.7214G > T	p.Trp2405Leu	M72, F73
18	c.8311G > A	p.Glu2771Lys	M60
8	c.9564C > G	p.Asn3188Lys	M45, F48

**Table 5:** Clinical characteristics of ADPKD patients according to genotype.

General description	Rate^a^
	n = 49^a^
Gender, M/F	32/17
Average age at the time of test (years)	31.1 (range, 20–63)
Finding renal cysts at less than 20 years old	12 (24.5%)
With a family history	44 (89.8%)
Hypertension	37 (75.5%)
%ESRD^b^	26.5%
Urolithiasis	5 (10.2%)
Family history of haemodialysis	20 (40.8%)
Family history of renal transplantation	3 (6%)
Extrarenal cysts	5 (10%)
Male infertility oligospermia	10 (31.2%)

^a^Data summarized from 49 probands.

^b^ESRD defined as transplant, dialysis, or Modification of Diet in Renal Disease (MDRD) GER < 10 mL/min.
