# Corrigendum: Identification of novel *PKD1* and *PKD2* mutations in a Chinese population with autosomal dominant polycystic kidney disease

**DOI:** 10.1038/srep21578

**Published:** 2016-02-23

**Authors:** Bei Liu, Song-Chang Chen, Yan-Mei Yang, Kai Yan, Ye-Qing Qian, Jun-Yu Zhang, Yu-Ting Hu, Min-Yue Dong, Fan Jin, He-Feng Huang, Chen-Ming Xu

Scientific Reports 5: Article number: 1746810.1038/srep17468; published online: 12032015; updated: 02232016

This Article contains typographical errors in Table 2.

For Family No. 19, the cDNA change ‘c.8795_8796insA’ and protein change ‘p.His2932GlnfsX5’ were incorrectly given as ‘c.8495-8496insA’ and ‘p.His2932GlnfsX4’ respectively.

For Family No. 21, the cDNA change ‘c.11436_11442dupCGTGTAT’ and protein change ‘p.Asp3815ArgfsX3’ were incorrectly given as ‘c.11433-11439dupCGTGTAT’ and ‘p.Ala3812ValfsX4’ respectively.

For Family No. 34, the cDNA change ‘c.12172delG’ and protein change ‘p.Val4058TrpfsX140’ were incorrectly given as ‘c.12169delG’ and ‘p.Val4057TrpfsX139’ respectively.

