# Supplementary material for: Identification of novel PKD1 and PKD2 mutations in a Chinese population with autosomal dominant polycystic kidney disease
Source: Sci Rep. 2015 Dec 3;5:17468. doi: 10.1038/srep17468 (PMC4668380; doi:10.1038/srep17468)
Supplement: Supplementary Information [file srep17468-s1.doc]

**Identification of novel *PKD1* and *PKD2* mutations in Chinese population with autosomal dominant polycystic kidney disease**

Bei Liu^1,2#^, Song-Chang Chen^3,4#^, Yan-Mei Yang^1,2^, Kai Yan^1,2^, Ye-Qing Qian^1,2^, Jun-Yu Zhang^3,4^, Yu-Ting Hu^3,4^, Min-Yue Dong^1,2^, Fan Jin^1,2^, Chen-Ming Xu^1,2,3,4*^

1. Women’s Hospital School of Medicine Zhejiang University, Hangzhou, Hangzhou 310006, P. R. China
2. Key Laboratory of Reproductive Genetics (Zhejiang University), Ministry of Education, Hangzhou 310006, P. R. China
3. Institute of Embryo-Fetal Original Adult Disease Affiliated to Shanghai Jiao Tong University School of Medicine, Shanghai 200030, P.R. China
4. The International Peace Maternity & Child Health Hospital Affiliated to Shanghai Jiao Tong University School of Medicine, Shanghai 200030, P. R. China

# These authors contribute equally to this study.

* Correspondence to: Dr. Chen-Ming Xu, Department of Reproductive Genetics, International Peace Maternity and Child Health Hospital, Shanghai Jiao Tong University School of Medicine, 910 Hengshan Road, Shanghai 200030, P.R. China; Phone: +86 21 64073897; Fax: +86 21 64078219; E-mail: [chenming_xu2006@163.com](mailto:chenming_xu2006@163.com)

**Table S1 polymorphisms**

| **Exon** | **cDNA change** | **Protein**  **change** | **Previous description** | **NB** |
| --- | --- | --- | --- | --- |
| *PKD1* |  |  |  |  |
| 10 | c.2039A > T | p.Y680F | PKDB | 2 |
| 15 | c.3868C >G | p.L1290V | PKDB | 1 |
| 15 | c.4340C > T | p.A1447V | PKDB | 1 |
| 15 | c.4810G> A | p.V1604M | PKDB | 1 |
| 15 | c.6070C>T | p.Arg2024Cys | [1] | 1 |
| 15 | c.6598C > T | p.R2200C | PKDB | 1 |
| 16 | c.7144C>T | p. A2312V | PKDB | 1 |
| 21 | c.7913A >G | p.H2638R | PKDB | 1 |
| 23 | c.8750C > T | P.A2917V | PKDB | 1 |
| 27 | c.9548G>A | p.R3183Q | PKDB | 1 |
| 35 | c.10529C >T | p.T3510M | PKDB | 6 |
| 36 | c.10678G> A | p.G3560R | PKDB | 3 |

Nb: number of probands carrying the sequence variant.

Reference: 1. Eisenberger, T. *et al.* An Efficient and Comprehensive Strategy for Genetic Diagnostics of Polycystic Kidney Disease. *PLoS One* **10,** e0116680 (2015).

**Table S2 LR primers for *PKD1* and *PKD2***

| **Fragment** | **Size (Kb)** | **Exon** | **Forward primer** | **Reverse primer** | **Tempreture (℃)** |
| --- | --- | --- | --- | --- | --- |
| PKD1 | | | | | |
| L1 | 2.2 | 1 | CCATCCACCTGCTGTGTGACCTGGTAAAT | CCACCTCATCGCCCCTTCCTAAGCAT | 68 |
| L2 | 4.6 | 2-7 | ATTTTTTGAGATGGAGCTTCACTCTTGCAGG | CGCTCGGCAGGCCCCTAACC | 68 |
| L3 | 4.2 | 8-12 | CCGCCCCCAGGAGCCTAGACG | CATCCTGTTCATCCGCTCCACGGTTAC | 68 |
| L4 | 4.4 | 13-15 | TGGAGGGAGGGACGCCAATC | GTCAACGTGGGCCTCCAAGT | 68 |
| L5 | 3.4 | 15-21 | AGCGCAACTACTTGGAGGCCC | GCAGGGTGAGCAGGTGGGGCCATCCTA | 70 |
| L6 | 0.3 | 22 | GAGGCTGTGGGGGTCCAGTCAAGTGG | AGGGAGGCAGAGGAAAGGGCCGAAC | 64 |
| L7 | 4.2 | 23-28 | CCCCGTCCTCCCCGTCCTTTTGTC | AAGCGCAAAAGGGCTGCGTCG | 68 |
| L8 | 5.8 | 29-34 | GGCCCTCCCTGCCTTCTAGGCG | GTTGCAGCCAAGCCCATGTTA | 68 |
| 35-37 | 0.7 | 35-37 | GGGATGAATTCACAGCCTAC | GGAGACAAGAGACGGAGGT | 62 |
| 38-40 | 1.1 | 38-40 | AAGCCCTGCTGTCACTGT | TACTCCCTTGTCCTTGGC | 56 |
| 41-43 | 1.1 | 41-43 | GGGAGTAGTTCTCCAGGAGTG | CGAGAAATCTGTCTGCTTGC | 62 |
| 44-46 | 1.1 | 44-46 | GGCTGCAAGCAGACAGATT | GCGGTGTCCACTCCGACTCC | 56 |
| *PKD2* | | | | | |
| 1-1 | 0.5 | 1 | AGAGGGAGGCGGGCCAAAGG | CGGGCGCCACTCTACGTCCA | 62 |
| 1-2 | 0.4 | 1 | GTGGAGCCGCGATAACCCCG | AGGCGGAACGCAGAGGGGAT | 62 |
| 2 | 0.5 | 2 | TTGTGCTTTATTTTCCCTTTTGCCA | TGCCTCTCCCGTCCTGTGTT | 59 |
| 3-4 | 2.5 | 3-4 | AGGGGAAAGGAAGGCAAGGGTGA | TGCCTTGGTGAAGGTGTCAGGGA | 65 |
| 5-6 | 4.0 | 5-6 | GCCAGGTCAGGCACAGTACCC | AGCGTGGCTGAGAGCATACTGT | 63 |
| 7-8 | 4.5 | 7-8 | TGGCAGGGCTTAACACTTTCCATTT | TCTTGAGAAGCAGTGACAACTCTGA | 65 |
| 9-10 | 4.7 | 9-10 | ACCGTGCCCAGCTTGTGTTT | CTGCCGTGGAAGGTCAAGGG | 65 |
| 11-13 | 2.9 | 11-13 | CCAGCACGTACTTGTTGAATGGCC | GGGAACTGCCTGGTCTCATGTGG | 65 |
| 14-15 | 1.0 | 14-15 | GCCAGTGGGGCTGAAAAGACA | AGCATCCTATGGTGGTCAGGGCA | 70 |

**Table S3 specific primers of *PKD2* for RT-PCR**

| **Primer** | **Sequence** |
| --- | --- |
| **PKD2-E1F** | GGAATGGTGGTGGAGATGGA |
| **PKD2-E2R** | TGAGTCTTGTTCCCCAGAGA |
| **PKD2-E4R** | TCGGTTGTCAGCTTCAGTCT |
| **PKD2-E13F** | CTCGAAGCCTGGATGACTCT |
| **PKD2-E14R** | GATCACGGCGTCAATCTTGG |
| **GAPDH-F** | TGATGACATCAAGAAGGTGGTGAAG |
| **GAPDH-R** | TCCTTGGAGGCCATGTGGGCCAT |

**
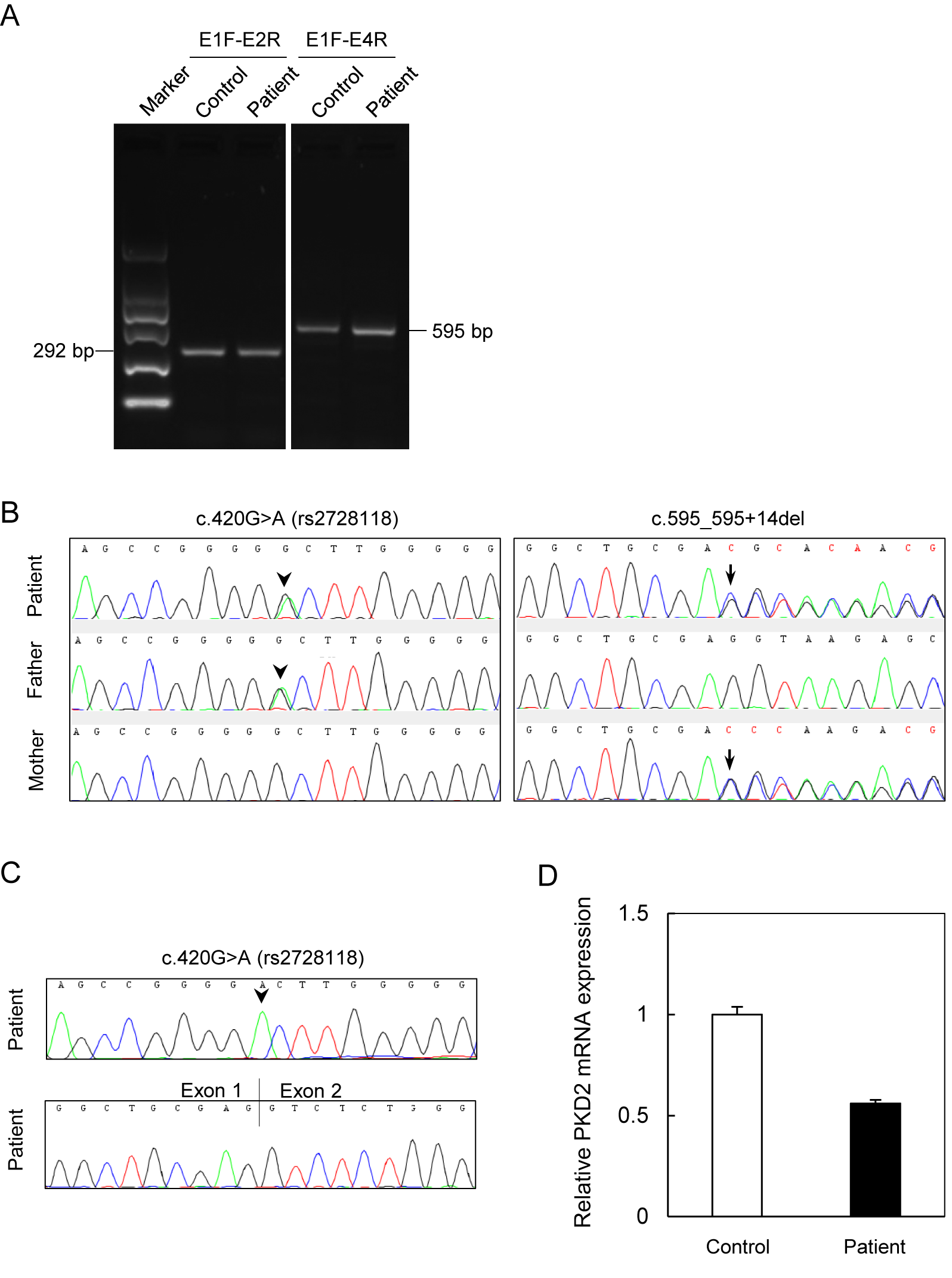
**

**Figure S1 (A)** Using reverse-transcriptase PCR primers in exon 1, exon 2 and exon 4, both the control and the patient showed a correct splicing pattern. bp, base pairs. **(B)** The patient carried a heterozygous SNP (c.420G>A; rs2728118) in the upstream of the c.595_595+14del mutation. **(C)** cDNA sequencing showed that the transcript of the patient was spliced correctly and homozygote for rs2728118 (AA). **(D)** The relative *PKD2* mRNA level of the patient was measured by qPCR using glyceraldehyde-3-phosphate dehydrogenase (*GAPDH*) gene as an internal control.


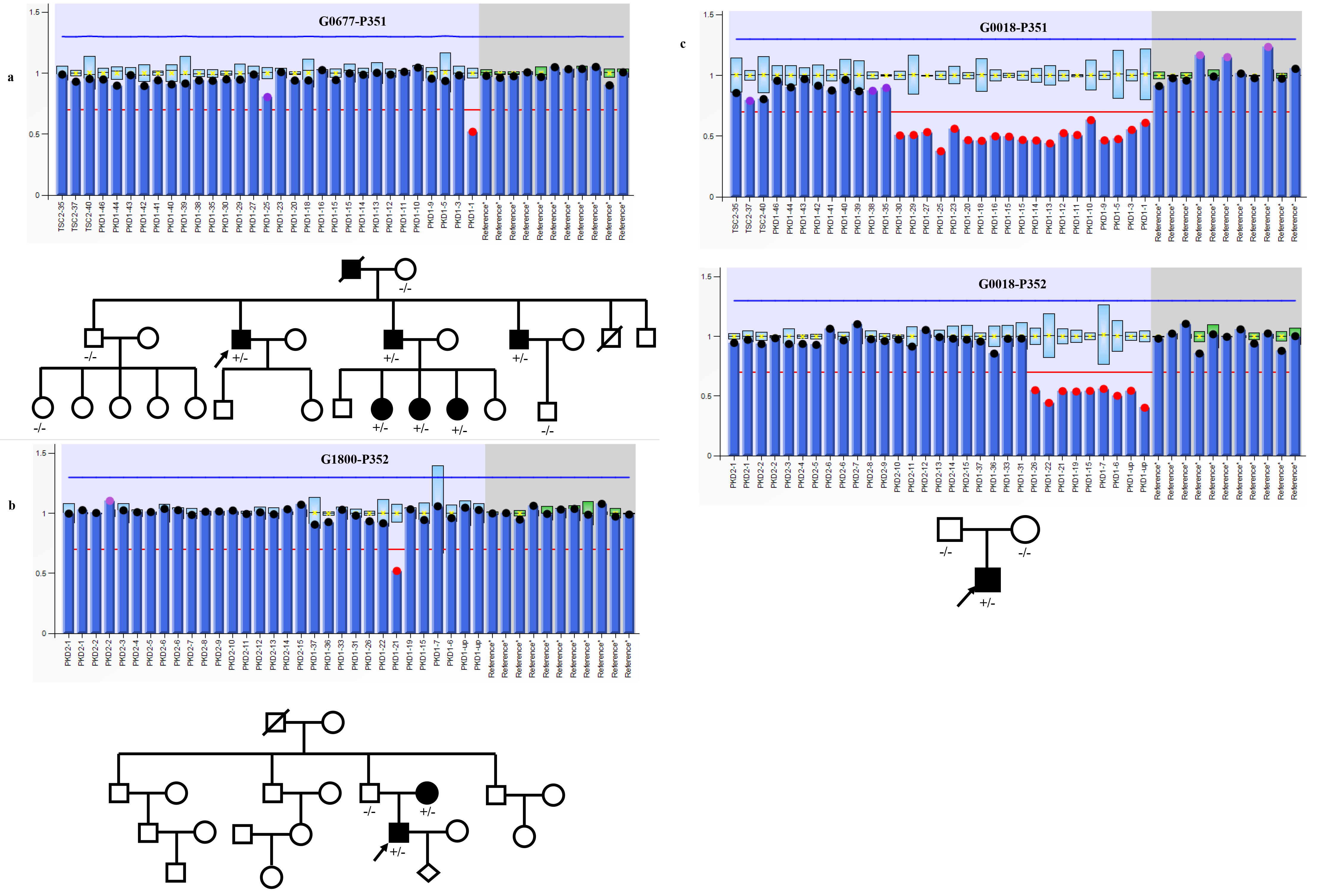


**Figure S2** MLPA data and pedigrees showing deletions of *PKD1* in three Chinese families of ADPKD. *PKD1* and reference probes are listed on the x axis. The relative peak area of the amplification product (peak ratio) is depicted on the y axis. The deletions of *PKD1* presented co-segregation with *PKD1* disease phenotype in these families. (a) The deletion of *PKD1* exon 1 was detected in patient G0677. (b) The deletion of *PKD1* exon 21 was detected in patient G1800. (c) The deletion of *PKD1* exon 1-30 was detected in patient G0018.
